# A Retrospective Study on the Aetiology of Clinical Bovine Mastitis and Its Antibiotic Resistance Profiles in Western Australia Dairy Farms

**DOI:** 10.3390/microorganisms14010254

**Published:** 2026-01-21

**Authors:** Hilary Chok, Michael Laurence, Joshua W. Aleri

**Affiliations:** 1 Midwest Vet Centre, 117 Cathedral Avenue, Geraldton, WA 6530, Australia; 2 College of Veterinary Medicine, Murdoch University, 90 South Street, Murdoch, WA 6150, Australia; 3 Meat Livestock Australia Level 1, 40 Mount Street, North Sydney, NSW 2060, Australia; 4 School of Veterinary Science, The University of Queensland, 5391 Warrego Highway, Gatton, QLD 4343, Australia

**Keywords:** antibiotic resistance, clinical mastitis, dairy cattle, Western Australia

## Abstract

Clinical data on antimicrobial profiles are useful for dairy udder health treatment programmes and represents a component of antimicrobial stewardship. The study aimed to determine the bacterial aetiology of clinical mastitis in dairy herds in Western Australia and to evaluate their antibiotic resistance profiles. This retrospective study utilised clinical antimicrobial profile data from two referral diagnostic centres within the region of Western Australia. A total of 545 mastitic samples were submitted for antimicrobial culture and testing over a period of 10 years (2008–2018). Of these, 406 showed bacterial growth and 139 no bacterial growth was observed. The most common isolates were *Streptococcus uberis* (25.3%), *Staphylococcus aureus* (17.2%), and *Escherichia coli* (9.4%). No growth was identified in 25.5% of the mastitis milk samples. The antimicrobial profiles revealed high susceptibilities towards cefuroxime (95.7%), clavulox (89.4%), and oxytetracycline (89%), whilst showing high resistance towards novobiovin (70%). From this study, it is concluded that there was a decline in the resistance trends towards the isolates of both *S. uberis* and *S. aureus* over the 10-year period and contagious mastitis had a higher occurrence. There is a need to consider surveillance programmes that determine the patterns of on-farm antimicrobial usage and further characterise the pathogens based on the presence of resistance antimicrobial genes. Data on antimicrobial surveillance represent an important component of antimicrobial stewardship.

## 1. Introduction

Mastitis is a significant and costly disease in dairy herds across many countries [1,2,3]. It has been reported to be the second most common reason for involuntary culling after reproductive issues [4,5,6]. According to Countdown 2020 published by Dairy Australia, the average cost to treat a single cow for mastitis was estimated to be $270 [7]. It was also estimated that Australian farmers lose more than $150 million per annum through poor udder health of their dairy cows [7]. Major costs reported include milk production losses, discarded milk, veterinary services, drugs, labour, and premature culling [8,9,10]. Apart from causing significant economic losses, mastitis also has negative impacts on an animal’s welfare and is one of the main reasons for antimicrobial use in dairy herds [11,12,13]. Antibiotics play a significant role in supporting and improving both human and animal health and welfare through the treatment of pathogens [14]. Despite the advocacy for responsible use and good stewardship on the use of antibiotics in veterinary medicine, there has been an increasing trend of antimicrobial resistance at the global level [15,16]. Focusing on the dairy industry, methicillin-resistant *S. aureus* (MRSA) strains were recently isolated from bovine intramammary infections (IMIs) cases in Argentina and India [17,18]. Good antimicrobial stewardship can greatly reduce the rate of antimicrobial resistance through prudent use of existing antibiotics and replacement of antimicrobial agents with non-antimicrobial alternatives, wherever possible [19,20,21]. There are limited data on the aetiology and antimicrobial profile of clinical mastitis-causing pathogens in Western Australia (WA). Clinical mastitis can be caused by contagious and environmental bacterial pathogens described elsewhere [22]. The transmission and spread of mastitis is caused by several animal-, managemental-, environmental-, and pathogen-level factors described elsewhere [22].

Studies on mastitis in dairy farms in the south-west region of Western Australia did characterise the bacterial pathogens associated with clinical mastitis and subclinical mastitis cases and their associated antimicrobial susceptibility profiles identified the need for ongoing surveillance programmes and pattern profiles of resistance genes among cases of mastitis [23]. Understanding the trends, patterns, and surveillance of resistance against various antibiotics is an important and integral component of good antimicrobial stewardship [24]. The aim of this study was to document the pathogens isolated from clinical mastitis cases within dairy herds in WA over the past 10 years and to evaluate their antimicrobial resistance profile. It is essential to undertake such surveillance to document trends as a reference point for future interventions.

## 2. Materials and Methods

### 2.1. Study Design, Area, and Sample Collection

A retrospective study was conducted based on culture and sensitivity results provided by Vetpath, a privately run diagnostic service, and the diagnostic laboratory of the Department of Primary Industries and Regional Development (DPIRDs), Western Australia, formerly the Department of Agriculture Food Western Australia (DAFWA). There is potential bias regarding the type of laboratory centre that processed a clinical sample, possibly due to the preference by the clinician or close proximity to the dairy farms. The milk samples were all new cases and were collected aseptically to avoid the risk of contamination. This included wearing gloves throughout the collection process, fore-stripping of the quarters prior to sample collection, sterile preparation, and drying of teats. A case of clinical mastitis was characterised by physical changes in the milk colour or consistency. Onset of clinical mastitis was first identified via clinical presentation on the udder and teats including swelling, heat, hardness, redness and pain of the udder, and physical changes in the milk. To ensure that accurate results were obtained for culture and sensitivity testing, the milk samples were collected prior to antimicrobial treatment. Approximately 3 to 5 mL of mastitic milk was collected into each collection vial and labelled with the date and animal identification number. The milk samples were transported to the respective laboratories at 4 °C.

### 2.2. Data Collection, Storage, and Analysis

A total of 260 milk samples were submitted to Vetpath between January 2014 and June 2018 for culture and sensitivity testing. These milk samples were tested for susceptibility against the following eight antibiotics: cefuroxime (CM), clavulox (CX), cloxacillin (CN), lincomycin (L), neomycin (NE), novobiocin (NO), penicillin (P), and oxytetracycline (OX).

A total of 285 mastitic milk samples were submitted to DPIRD between 2008 and 2018 for culture and sensitivity testing. At DPIRD, the milk samples were tested for susceptibility against the following ten antibiotics: clavulox (CX), cloxacillin (CN), lincomycin (L), neomycin (NE), novobiocin (NO), penicillin (P), oxytetracycline (OX), oleandamycin (OL), tylosin (T), and cephalothin (CE).

The two sets of data from Vetpath and DPIRD were combined into one Excel spreadsheet (a total of 545 isolates) and were analysed together. Descriptive statistics (specifically measures of frequency) were used to analyse the isolate and culture and sensitivity test results submitted over the years. *S. uberis*, *S. aureus,* and non-haemolytic *E. coli* were analysed for their resistance profiles over multiple years.

### 2.3. Culture and Identification

Briefly, in both diagnostic centres, all milk samples were cultured on horse blood agar (HBA), Columbia naladixic acid (CNA), and MacConkey II agar; a Gram stain was performed on each sample. The HBA and CNA agar plates were incubated overnight at 37 °C in 5% carbon dioxide, whereas the MacConkey II agar plates were incubated at 37 °C in oxygen [25]. If growth was present after overnight culture, the bacterial colonies were speciated and tested for susceptibilities against different types of antibiotics.

Bacteria species were identified using biochemical methods including catalase, oxidase, indole and tube coagulase tests, chromogenic agars CPS and mannitol salt agar (MSA), and kit tests such as *Streptococcus* spp. grouping. A Vivek 2 compact analyser (bioMérieux, Marcy l’Etoile, France) was used to identify bacteria that could not be identified via biochemical methods as it contains a bigger range of biochemical tests. *Streptococcus* spp. often cannot be identified using the Vivek 2 (v8.01) compact analyser; thus, an API rapid ID strep kit was used if both methods previously mentioned failed to identify the bacterial isolate.

Inoculates were distributed onto Mueller–Hinton (MH) agars and antibiotic discs were placed on top of it. After incubation overnight at 37 °C, zone sizes were measured, and susceptibilities were determined according to CSLI guidelines [26].

## 3. Results

From the 545 mastitic milk samples submitted, 22 different pathogens (cases) were isolated (Table 1), of which *S. uberis* had the highest occurrence (138 cases, 25.3%). It was followed by *S. aureus* (94 cases, 17.2%); *E. coli* (51 cases, 9.4%); coagulase-negative *Staphylococcus* spp. (31 cases, 5.7%); *Streptococcus dysgalactiae* (16 cases, 2.94%); other *Streptococcus* spp. (15 cases, 2.75%); *Enterobacter* spp. (10 cases, 1.83%); *Klebsiella* spp. (8 cases, 1.47%); *Serratia* spp. (8 cases, 1.5%); and *Bacillus* spp. (5 cases, 0.92%), with a few cases (15) of minor pathogens. No growth was observed in 139 (25.5%) of the mastitic milk samples.

All the pathogens cultured from the milk samples were tested against the antimicrobial agents listed in Table 2. The isolates were most susceptible to cefuroxime (95.7%), followed by clavulox (89.4%), oxytetracycline (89%), cloxacillin (70.9%), and penicillin (70%). On the other hand, the highest level of resistance among the isolates was against novobiocin (70%), followed by oleandamycin (34.3%) and neomycin (28.6%).

In vitro susceptibility and resistance patterns of all the isolates are shown in Table 3 and Table 4. *S. uberis* showed complete susceptibility (100%) towards cefuroxime, cephalothin, oleandamycin, and tylosin, while having moderate to good susceptibility towards the remaining antibiotics. *S. aureus* isolates’ susceptibility patterns were similar to *S. uberis*. All the antibiotics, except for novobiocin and oleandamycin, were shown to be quite effective against *S. aureus*, with susceptibility levels above 80%. Novobiocin, however, was very ineffective, with a susceptibility level of only 4%. *E. coli* had similar susceptibility and resistance patterns towards the antimicrobial agents.

Some of the less frequently cultured bacteria such as *Acinetobacter* spp., *Aerococcus* spp., and *Aeromonas* spp. were demonstrated to be resistant towards all the antimicrobial agents tested in this study. In contrast, isolates including *Histophilus somni*, *Leclercia adecarboxylata*, *Pantoea* spp., *Pseudomonas* spp., and *Sphingomonas paucimobilis* were susceptible to all the antimicrobial agents except for novobiocin.

**Figure 1:**
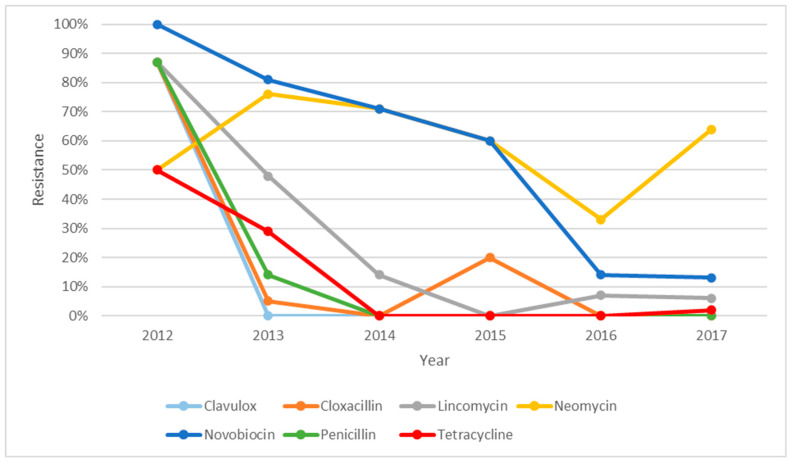
Resistance profile of *S. uberis* from clinical mastitic samples obtained from dairy farms in Western Australia, as observed from 2008 to 2018, from two diagnostic centres.

**Figure 2:**
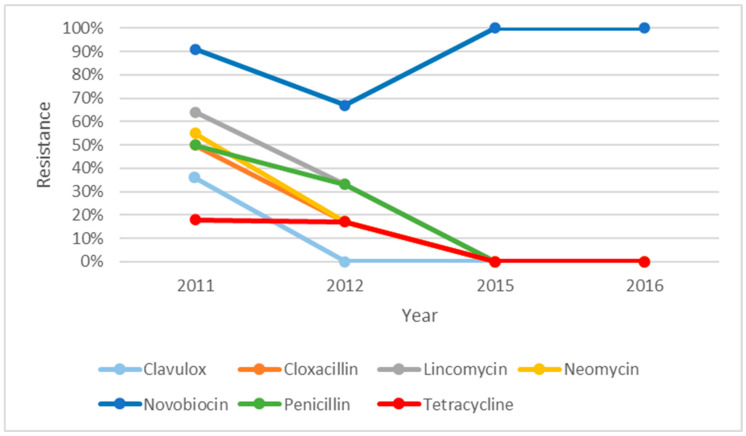
Resistance profile of *S. aureus* from clinical mastitic samples obtained from dairy farms in Western Australia, as observed from 2008 to 2018, from two diagnostic centres.

**Figure 3:**
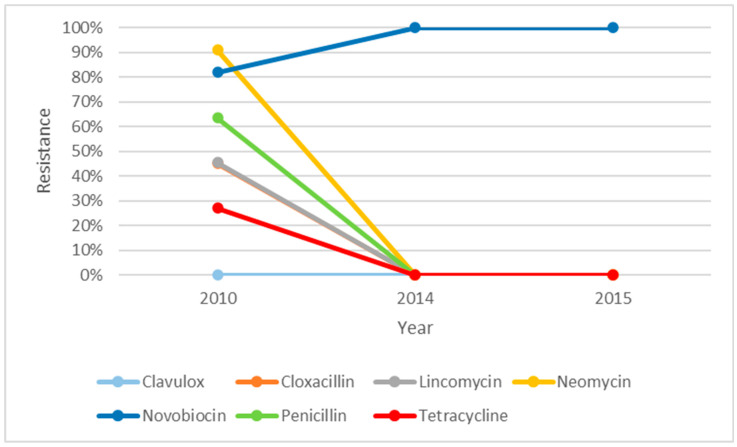
Resistance profile of non-haemolytic *E. coli* over 2010, 2014, and 2015 from clinical mastitic samples obtained from dairy farms in Western Australia, as observed from 2008 to 2018, from two diagnostic centres.

The resistance profile of *S. uberis* over five years, i.e., from 2012 to 2017, is illustrated in Figure 1. There were descending trends of resistance for all the antibiotics over five years except for neomycin. The resistance against neomycin was seen to be decreasing from 2013 but increased to 65% in 2017. There was an increase in resistance against cloxacillin in 2015; however, it dropped to zero in 2016. Figure 2 shows the resistance profile of *S. aureus* over four years (2011, 2012, 2015, and 2016). Novobiocin was the only antibiotic that displayed a different trend from the other antibiotics. The percentage of isolates that were resistant towards novobiocin seemed to decrease between 2011 and 2012 but changed to 100% resistance in 2015 and stayed at 100% in 2016. The *S. aureus* isolates were shown to have steady reduction in resistance towards all the other antibiotics and were zero in 2015 and 2016. As shown in Figure 3, non-haemolytic *E. coli* showed variable resistance towards all the antibiotics tested except for clavulox; however, it tapered off to no resistance in 2014 and 2015. This was an exception for novobiocin, which showed increased resistance over the years.

## 4. Discussion

Antimicrobial surveillance is an important component in dairy udder health programmes and in antimicrobial stewardship. The objectives of this study were to identify pathogens isolated from clinical mastitic samples and to evaluate their antimicrobial resistance profiles. Generally, *S. uberis* was the most common isolate recovered, followed by *S. aureus*. All the isolates demonstrated high susceptibilities towards cefuroxime, but they showed high resistance towards novobiovin (70%). A limitation to this study was the number of cows included in the analysis. The small sample size and lack of information on the geographical distribution of the mastitis cases across the regions are acknowledged as additional limitations of the study. Despite these limitations, the findings from this study provide a valuable insight into the occurrence and aetiology of mastitis cases and antibiotic resistance profile for *S. aureus* and *S. uberis* over the years in the Western Australian dairy industry.

In our study, *S. uberis* was the most common isolate cultured (34%) from the mastitic milk samples. This is consistent with the findings in other pasture-based systems [27,28] but contrary to the findings of other studies [23]. Despite its reputation as an environmental pathogen, *S. uberis* also has the potential to cause contagious mastitis and could be spread from cow to cow during milking [29,30]. High prevalence of environmental pathogens usually reflects managemental issues within dairy farms such as poor bedding management, overcrowding, and access to muddy areas [31]; however, this is more commonly seen in systems where dairy cattle are intensely managed or housed. In a pasture-based system, which is more common in Australia, attention to management practices plays an important role to reduce cow-to-cow transmission of contagious *Streptococcus* spp. [30].

*S. aureus*, the second most frequently isolated pathogen, is a contagious pathogen and can produce different types of enzymes and toxins that can evade tissue and eventually damage the mammary tissue. It is also known to be challenging to treat as it can survive and form biofilms within fibrous tissue of teat canals, where antibiotics cannot effectively penetrate [13,32]. Furthermore, it is becoming harder for antibiotics to cure mastitis caused by *S. aureus* as a lot of the strains have acquired the ability to produce beta-lactamase enzymes that could deactivate antibiotics like penicillin [33]. Due to its ability to survive in fibrous tissue and resist against some types of antibiotics, *S. aureus* has the tendency to cause chronic IMI, which could eventually become untreatable [34]. This reiterates the importance of identifying the aetiology of IMI and determining its susceptibility so that appropriate treatments can be given.

A total of 139 (25.5%) milk samples in our study yielded no growth. This is comparable with the percentage of culture-negative samples detected in other studies [35,36]. Possible reasons include low concentration of bacteria in the sample, improper sample handling and transport, inability of pathogens to grow in standard culture media, and interference from antibiotics or disinfectants [37,38]. A high proportion of culture-negative milk samples collected from cows showing IMI clinical signs can be significant, as identification of causative pathogens is crucial to the selection of effective antimicrobial treatments for the condition. It is important to bear in mind that *Mycoplasma bovis* and other related *Mycoplasma* spp. could be suspected of involvement in IMI; however, they are not routinely cultured from mastitis samples unless specifically requested. Recent studies within the dairying region of Western Australia showed a high seroprevalence of mycoplasma in dairy farms [39]. There is a need for surveillance for *Mycoplasma* in mastitis cases.

The results of this study will support aspects of antimicrobial stewardship in the dairying region in Western Australia. The Australian Veterinary Association (AVA) has urged veterinarians to choose first-line antibiotics, including tetracyclines, sulphonamides, and beta-lactams, for IMI treatments, as part of good antimicrobial stewardship practice [40]. According to the susceptibility profile generated in the study, these first-line antibiotics were proven to maintain their efficacies against common IMI pathogens. Cefuroxime and Clavulox showed the highest susceptibility levels among all the isolates and this is most likely due to their rare use in production animals. On the other hand, novobiocin was shown to be highly ineffective in treating IMI. Fujimoto-Nakamura et al. (2005) [41] suggested that the accumulation of mutant genes in *Staph aureus* is a factor leading to high-level resistance towards novobiocin. Commercially, novobiocin has been sold in combination with penicillin to increase its spectrum of action [42,43]. Alternative therapies for IMI have been suggested, including non-antimicrobial drug therapies (such as oxytocin) and pre-milking teat disinfection; however, they are less effective compared to aggressive antimicrobial treatments [44,45]. As incidence of IMI is influenced by factors such as management, treatment of clinical cases, stock replacement policy, dry-cow therapy and teat disinfection, alternative therapies, including improved management and husbandry, they might need to be used in conjunction with antimicrobial treatments to achieve the best therapeutic results.

## 5. Conclusions

This study described the aetiology and frequency of clinical mastitis-causing pathogens, with their antibiotic resistance profile over 10 years within dairy herds in Western Australia. The findings of the study are limited to the geographical location, nature of the retrospective study design, missing data points, as well as data sets such as the number of isolates within each milk sample. Resistance trends for the three major mastitis-causing pathogens (*S. uberis*, *S. aureus,* and non-haemolytic *E. coli*) were demonstrated to decline over the years. There is a need for continued surveillance programmes to determine the epidemiology of on-farm antimicrobial usage and further characterise the pathogens based on the presence of resistance genes.

## Figures and Tables

**Table 1:** Frequency and occurrences of the isolated pathogens from clinical mastitic samples obtained from dairy farms in Western Australia, as observed from 2008 to 2018, from two diagnostic centres.

Bacteria Isolated	Frequency	Prevalence (%)
No growth	139	25.5
*Streptococcus uberis*	138	25.3
*Staphylococcus aureus*	94	17.2
*Escherichia coli*	51	9.4
*Coagulase-negative Staphylococcus* spp.	31	5.7
*Streptococcus dysgalactiae*	16	2.94
Other *Streptococcus* spp.	15	2.75
*Enterobacter* spp.	10	1.83
*Klebsiella* spp.	8	1.47
*Serratia* spp.	8	1.5
*Bacillus* spp.	5	0.92
*Lactococcus* spp.	4	0.73
*Sphingomonas paucimobilis*	4	0.73
*Acinetobacter* spp.	3	0.55
*Corynebacterium* spp.	3	0.55
*Enterococcus* spp.	3	0.55
*Leclercia adecarboxylata*	3	0.55
*Pantoea* spp.	3	0.55
*Aeromonas* spp.	2	0.37
*Pseudomonas* spp.	2	0.37
*Aerococcus viridians*	1	0.18
*Histophilus somni*	1	0.18
*Shewanella putrefaciens*	1	0.18
**Total**	**545**	**100**

**Table 2:** In vitro antimicrobial susceptibility test results of all mastitic milk isolates (545) obtained from dairy farms in Western Australia, as observed from 2008 to 2018, from two diagnostic centres.

Antibiotics	Sensitive	Resistant	No Susceptibility Recorded
Cefuroxime (CM)	247 (95.7%)	11 (4.3%)	0 (0%)
Clavulox (CX)	363 (89.4%)	35 (8.6%)	8 (2.0%)
Cloxacillin (CN)	288 (70.9%)	73 (18%)	45 (11.1%)
Lincomycin (L)	264 (65%)	96 (23.6%)	46 (11.4%)
Neomycin (NE)	200 (49.3%)	116 (28.6%)	90 (22.1%)
Novobiocin (NO)	122 (30%)	284 (70%)	0 (0%)
Penicillin (P)	284 (70%)	75 (18.5%)	47 (11.5%)
Oxytetracycline (OX)	361 (89%)	45 (11%)	0 (0%)
Cephalothin (CP)	118 (80.8%)	28 (19.2)	0 (0%)
Oleandamycin (OL)	95 (65%)	50 (34.3%)	1 (0.7%)
Tylosin (T)	110 (75.3%)	34 (23.3%)	2 (1.4%)

**Table 3:** In vitro susceptibility of the bacterial isolates from clinical mastitic samples obtained from dairy farms in Western Australia, as observed from 2008 to 2018, from two diagnostic centres.

	Responses to Application of Antimicrobial Disc (Susceptibility in %)											
Isolates	No.	CM	CX	CN	L	NE	NO	P	OX	CP	T	OL
*Acinetobacter* spp.	3	0	0	0	0	0	0	0	0	-	-	-
*Aerococcus* spp.	1	0	0	0	0	0	0	0	0	-	-	-
*Aeromonas* spp.	2	0	0	0	0	0	0	0	0	-	-	-
*Bacillus* spp.	5	0	80	80	20	0	60	60	80	0	0	0
*Corynebacterium* spp.	3	100	100	0	0	0	0	0	0	-	-	-
*Enterobacter* spp.	10	100	100	10	0	60	0	50	80	0	0	0
*Enterococcus* spp.	3	100	100	33	67	100	33	100	100	0	0	0
*Escherichia coli*	51	100	96	86	88	76	8	82	94	0	0	0
*Histophilus somni*	1	100	100	100	100	100	0	100	100	-	-	-
*Klebsiella* spp.	8	100	100	62	50	38	25	62	100	29	0	0
*Lactococcus* spp.	4	100	75	50	50	50	0	50	100	100	0	0
*Leclercia adecarboxylata*	3	100	100	100	100	100	0	100	100	-	-	-
*Pantoea* spp.	3	100	100	100	100	100	0	100	100	-	-	-
*Pseudomonas* spp.	2	100	100	100	100	100	0	100	100	-	-	-
*Serratia* spp.	8	100	100	87.5	75	50	0	87.5	100	100	100	0
*Shewanella putrefaciens*	1	-	100	0	0	0	0	0	100	100	100	0
*Sphingomonas paucimobilis*	4	100	100	100	100	100	0	100	100	-	-	-
*Staphylococcus aureus*	94	100	91.2	87	82	86	4	86	95	100	100	61
*CNS*	31	100	74	58	61	61	3	58	61	100	100	100
*Streptococcus dysgalactiae*	16	100	94	87.5	75	50	44	75	94	100	100	100
*Streptococcus uberis*	138	100	88	61	51	14	67	59	92	100	100	100
Other *Strep* spp.	15	100	100	87	73	27	40	73	87	100	100	100

**Table 4:** In vitro resistance of the bacterial isolates from clinical mastitic samples obtained from dairy farms in Western Australia, as observed from 2008 to 2018, from two diagnostic centres.

	Responses to Application of Antimicrobial Disc (Resistance in %)											
Isolates	No.	CM	CX	CN	L	NE	NO	P	OX	CP	T	OL
*Acinetobacter* spp.	3	100	100	100	100	100	100	100	100	-	-	-
*Aerococcus* spp.	1	100	100	100	100	100	100	100	100	-	-	-
*Aeromonas* spp.	2	100	100	100	100	100	100	100	100	-	-	-
*Bacillus* spp.	5	100	20	20	80	100	40	40	20	100	100	100
*Corynebacterium* spp.	3	0	0	100	100	100	100	100	100	-	-	-
*Enterobacter* spp.	10	0	0	90	100	40	100	50	20	100	100	100
*Enterococcus* spp.	3	0	0	67	33	0	67	0	0	100	100	100
*E. coli*	51	0	4	14	12	24	92	18	6	100	100	100
*Histophilus somni*	1	0	0	0	0	0	100	0	0	-	-	-
*Klebsiella* spp.	8	0	0	38	50	62	75	38	0	71	100	100
*Lactococcus* spp.	4	0	25	50	50	50	100	50	0	0	100	100
*Leclercia adecarboxylata*	3	0	0	0	0	0	100	0	0	-	-	-
*Pantoea* spp.	3	0	0	0	0	0	100	0	0	-	-	-
*Pseudomonas* spp.	2	0	0	0	0	0	100	0	0	-	-	-
*Serratia* spp.	8	0	0	12.5	25	50	100	12.5	0	0	0	100
*Shewanella putrefaciens*	1	-	0	100	100	100	100	100	0	0	0	100
*Sphingomonas paucimobilis*	4	0	0	0	0	0	100	0	0	-	-	-
*Staphylococcus aureus*	94	0	8.8	13	18	14	96	14	5	0	0	39
*CNS*	31	0	26	42	39	39	97	42	39	0	0	0
*Streptococcus dysgalactiae*	16	0	6	12.5	25	50	56	25	6	0	0	0
*Streptococcus uberis*	138	0	5	7	15	28	33	7	8	0	0	0
Other *Strep* spp.	15	0	0	13	27	33	60	2	13	0	0	0

## Data Availability

The original contributions presented in this study are included in the article. Further inquiries can be directed to the corresponding author.
